# Implementation of an education session on buprenorphine induction in the emergency department, a resident-led initiative

**DOI:** 10.1186/s12954-023-00917-4

**Published:** 2024-01-28

**Authors:** Cara Marie Borelli, Han Tony Gao

**Affiliations:** 1 Yale University School of Medicine, New Haven, USA; 2 UT Health San Antonio, San Antonio, USA

**Keywords:** Buprenorphine, Opioid use disorder, Emergency medicine, Buprenorphine induction

## Abstract

**Introduction:**

Many physicians including emergency medicine physicians report insufficient training and education on prescribing buprenorphine for opioid use disorder. As emergency departments implement buprenorphine induction protocols, educational sessions can provide physicians with further familiarity with the treatment of opioid use disorder. This quality improvement project aimed to address the barrier of physician education in the implementation of buprenorphine initiation in the emergency department and presents a model for resident-led education sessions of emergency medicine physicians.

**Methods:**

The project was a resident-led educational quality improvement project on educating members of the Department of Emergency Medicine on buprenorphine induction. The thirty-minute educational session included a pre-test survey, lecture, practice case workshop, questions, post-test survey, and a discussion. The survey questions were designed for physicians including residents and faculty, but medical students were invited to complete the session.

**Results:**

Physicians including faculty and resident physicians responded positively to the educational survey, with an increase from 42.5 to 100% responding that they understood the risks and benefits of prescribing buprenorphine in the emergency department pre and post-survey respectively. Based on post-survey results, 88.5% of physicians responded that they planned to prescribe buprenorphine in the emergency department for patients meeting clinical criteria after completing the educational session.

**Conclusion:**

The results suggest that a resident-led training session can encourage peer involvement in buprenorphine induction to treat opioid use disorder in the emergency department.

## Introduction

Opioid use disorder has a high morbidity and mortality, and the emergency department has the potential to provide a critical role in the treatment of opioid use disorder beyond treating acute overdoses and medical sequelae of opioid use such as soft-tissue infections. Medications that address the underlying opioid use disorder include methadone, naltrexone intramuscularly, and buprenorphine, although pharmacologic and regulatory limitations with methadone and naltrexone make buprenorphine an ideal medication for initiation from the emergency department [1]. Buprenorphine is a long-acting, high-affinity partial agonist of the μ-opioid receptor used in the treatment of opioid use disorder. Buprenorphine is an effective medication for opioid use disorder [2, 3]. In one study, buprenorphine was associated with a reduced twelve-month risk of opioid-related mortality, with a reduced risk of 38 percent based on an adjusted hazard ratio of 0.62 [3]. Multiple regimens have been suggested for emergency department initiation of buprenorphine for opioid withdrawal including a standard-dose induction pathway [4], which was the topic of this workshop, as well as a high-dose induction pathway [5] and a low-dose induction pathway [6].

The implementation of opioid-agonist therapy for opioid use disorder in the emergency department has varied across the United States in both community and academic-based emergency departments [7]. Barriers to prescribing buprenorphine include lack of training and education, insufficient peer support and institutional support, individual stigma, lack of follow-up care, regulations surrounding obtaining the waiver, and reimbursement concerns [7]. The majority of physicians report a lack of training and education to be a barrier to prescribing buprenorphine [7].

Before the DEA DATA (X) waiver requirement was removed in January of 2023 (SAMHSA 8), a previous successful resident-physician-led initiative included a campaign to increase the percentage of resident physicians and faculty who had a DEA X-waiver (Martin 9) in a healthcare system that had a robust pre-existing bridge program to arrange for follow-up. A similarly formatted 1-h educational workshop was administered by faculty to first-year emergency medicine residents and medical students including a 30-min didactic session followed by a case-based session; in the workshop, pre and post-session workshop scores on tests were compared, and the average scores increased from 44 to 89% (*p* < 0.0001 using a paired, two-tailed *t*-test) [10]. This quality improvement project in the format of a resident-led workshop aimed to address the barrier of physician education in the implementation of buprenorphine initiation in the emergency department (Table 1).

**Table 1:** Demographics of the participants

Training level of participant	Number of participants, pre-lecture survey	Number of participants, post-lecture survey
Medical student	13	11
Resident physician	24	21
Faculty physician	9	5
Other (e.g.,, clinical pharmacist)	1	0
Total	47	37

## Methods

The project was a resident-led educational quality improvement project on educating members of the Department of Emergency Medicine on buprenorphine induction for opioid use disorder. The Project followed the Plan-Do-Study-Act design in order to provide a framework for an evidence-based didactic session for continued education for future trainees [11].

### Participants

The participants included medical students, emergency medicine residents, and emergency medicine faculty who were participating in the educational session as a component of weekly didactics for residents in an emergency medicine residency training program in San Antonio, Texas, in 2019. Emergency-medicine-trained clinical pharmacists who worked in the department were also invited to attend weekly didactics.

### Workshop format

The thirty-minute educational session began with a pre-workshop survey followed by a lecture on the pharmacology of buprenorphine, buprenorphine induction in the emergency department focusing on a standard-dose induction protocol, identifying opioid withdrawal, adjuncts for opioid withdrawal, and local resources for coordinating care for after discharge. After the lecture, there was a practice case workshop, multiple-choice questions to assess understanding in a discussion-based format, and a post-test survey. The lecture, workshop, and questions included information adapted from two emergency medicine didactic workshops on buprenorphine induction [10, 12]. The surveys were anonymous and accessed online by a QR code at the beginning and end of the education session. The surveys included one demographic question regarding the level of training and three Likert-scale questions (Table 2). This training was primarily intended as a resident-to-resident training session to encourage peer involvement in treating opioid use disorder, although there was a strong faculty interest in the workshop. As the training occurred during the weekly emergency medicine resident didactics, medical students who were completing their assigned rotation in emergency medicine were also invited to attend the workshop. However, two of the three survey questions presupposed medication prescribing ability in the emergency department, which did not apply to medical students based on their level of training.

**Table 2:** Physician responses (resident physicians and faculty physician) (*n* = 33 pre-session, *n* = 26 post-session)

Survey question	Percentage who agree or strongly agree: pre-session (*n* = 47) (%)	Percentage who agree or strongly agree: post-session (*n* = 37) (%)
“I understand the risks and benefits of prescribing buprenorphine in the emergency department”	42.4	100
“I would feel comfortable prescribing buprenorphine in the emergency department”	33.3	100
“I plan to prescribe buprenorphine in the emergency department for patients meeting clinical criteria”	48.5	88.5

### Data analysis

The pre-survey versus post-survey data were aggregated and analyzed as continuous data [13] based on the Likert scale with 1 corresponding with strongly disagree through 5 corresponding with strongly agree. Results were reported for all survey responses (from medical students, resident physicians, and faculty) as well as the following subgroups: physician-only responses (resident physicians and faculty), and medical-student-only responses. The results for each of the three individual questions were also analyzed separately for the subgroups.

## Results

Of the attendees of the session, 47 completed the pre-lecture survey and 37 completed the post-lecture survey. When the pre-survey versus post-survey data for all surveyed participants (medical students, resident physicians, faculty, and other) were aggregated for all three questions, the mean score pre-survey was 2.97 (standard deviation [SD] 1.16) and the mean score post-survey was 4.29 (SD 0.68), *p*-value < 0.0001 using a one-tailed *t*-test. When medical student scores were removed from the survey responses, the mean score pre-survey was 3.27 (SD 1.05), and the mean score post-survey was 4.42 (SD 0.57), *p*-value < 0.0001.

**Fig. 1:**
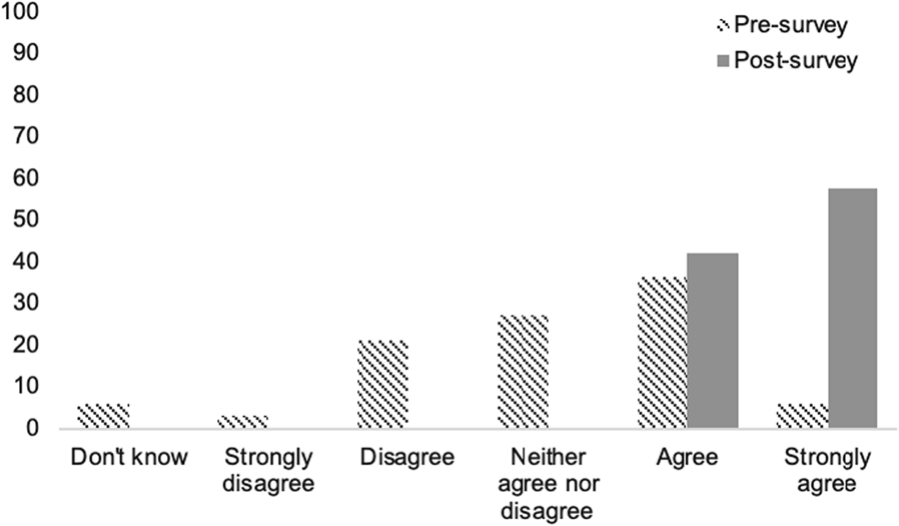
Percentage of physicians (resident physicians and faculty physician) (y-axis) with responses to the question “*I understand the risks and benefits of prescribing buprenorphine in the emergency department”* on a Likert scale (x-axis) (*n* = 33 pre-session, *n* = 26 post-session)

Physicians including faculty and resident physicians showed an increase from 42.5 to 100% responding that they understood the risks and benefits of prescribing buprenorphine in the emergency department pre and post-survey respectively (Table 2, Fig. 1). Based on post-survey results, 88.5% of physicians responded that they planned to prescribe buprenorphine in the emergency department for patients meeting clinical criteria after completing the educational session (Table 2).

The results from the survey questions applicable to medical students showed an increase from 7.6 to 100% of medical students responding that they understood the risks and benefits of prescribing buprenorphine in the emergency department pre and post-survey respectively (Table 3).

**Table 3:** Medical student responses (*n* = 13 pre-session, *n* = 11 post-session)

Survey question	Percentage who agree or strongly agree: pre-session (*n* = 47)	Percentage who agree or strongly agree: post-session (*n* = 37)
“I understand the risks and benefits of prescribing buprenorphine in the emergency department”	7.6%	100%

## Discussion

Barriers to prescribing buprenorphine include lack of training and education, insufficient peer support and institutional support, individual stigma, lack of follow-up care, regulations surrounding obtaining the waiver, and reimbursement concerns [7]. The majority of physicians report a lack of training and education to be a barrier to prescribing buprenorphine [7].

This education session demonstrably fulfilled its goal of increasing clinician training and education as evidenced by the data above. Although the session was aimed primarily at physicians, the inclusion of medical students helped to provide early educational familiarity with buprenorphine to supplement a curriculum with few educational opportunities in opioid use disorder. The results from the survey questions applicable to medical students were encouraging, with an increase from 7.6 to 100% of medical students responding that they understood the risks and benefits of prescribing buprenorphine in the emergency department pre and post-survey respectively.

Physicians including faculty and resident physicians also responded positively to the educational survey, with an increase from 42.5 to 100% responding that they understood the risks and benefits of prescribing buprenorphine in the emergency department pre and post-survey respectively. Based on post-survey results, 88.5% of physicians responded that they planned to prescribe buprenorphine in the emergency department for patients meeting clinical criteria after completing the educational session. Limitations exist in the gap between intention and action taken, but the results are encouraging in addressing one of the main barriers to treating opioid use disorder in the emergency department.

In addition to this educational goal, this session also may address the barrier of stigma. As described by Martin et al., a resident-led buprenorphine initiative can help to provide social support to physicians prescribing buprenorphine [9]. Evidence toward increased peer support for the initiative included motivation to begin buprenorphine in the emergency department group discussions as well as positive anonymous comments on the surveys. A future version of the workshop can include monitoring changes in physicians initiating buprenorphine induction before and after the workshop. Future implementations of this workshop are planned for incoming classes of emergency medicine residents in the residency training program where the workshop was initially given. The authors would also like to expand access to the workshop by providing the materials online for those who wish to lead in-person workshops at their institutions. Additional improvements to this workshop can emphasize encouraging interdisciplinary involvement to ensure that all members of the emergency medicine healthcare team are knowledgeable on the topic of buprenorphine induction.

Overall, a resident-physician-led educational session was associated with an increase in physician understanding and familiarity with initiating buprenorphine treatment in the emergency department. As emergency departments across the nation initiate buprenorphine protocols, resident-led education sessions may serve to increase clinician training and education as well as serve as a forum for discussing opioid use disorder treatment in the emergency department.

## Data Availability

Yes. Please provide a data availability declaration in the manuscript. Please refer to the submission guidelines for more information: The data supporting this study are available upon request to the corresponding author.

## References

[1] Hawk K, Hoppe J, Ketcham E, LaPietra A, Moulin A, Nelson L, Schwarz E, Shahid S, Stader D, Wilson MP, D'Onofrio G (2021). Consensus recommendations on the treatment of opioid use disorder in the emergency department. Ann Emerg Med.

[2] Mattick RP, Breen C, Kimber J, Davoli M (2014). Buprenorphine maintenance versus placebo or methadone maintenance for opioid dependence. Cochrane Database Syst Rev..

[3] Larochelle MR, Bernson D, Land T (2018). Medication for opioid use disorder after nonfatal opioid overdose and association with mortality: a cohort study. Ann Intern Med.

[4] D’Onofrio G, O’Connor PG, Pantalon MV (2015). Emergency department-initiated buprenorphine/naloxone treatment for opioid dependence: a randomized clinical trial. JAMA.

[5] Herring AA, Vosooghi AA, Luftig J, Anderson ES, Zhao X, Dziura J, Hawk KF, McCormack RP, Saxon A, D’Onofrio G (2021). High-dose buprenorphine induction in the emergency department for treatment of opioid use disorder. JAMA Netw Open.

[6] Moe J (2020). Microdosing and standard-dosing take-home buprenorphine from the emergency department: a feasibility study. J Am Coll Emerg Phys Open.

[7] Haffajee RL, Bohnert ASB, Lagisetty PA (2018). Policy pathways to address provider workforce barriers to buprenorphine treatment. Am J Prev Med.

[8] SAMHSA. Removal of data waiver (X-waiver) requirement. Removal of DATA Waiver (X-Waiver) requirement. https://www.samhsa.gov/medications-substance-use-disorders/removal-data-waiver-requirement.

[9] Martin A, Kunzler N, Nakagawa J, Lee B, Wakeman S, Weiner S, Raja AS (2019). Get waivered: a resident-driven campaign to address the opioid overdose crisis. Ann Emerg Med.

[10] Jennings L, Warner T, Bacro-Duverger B (2020). Identification and treatment of opioid withdrawal and opioid use disorder in the emergency department. MedEdPORTAL.

[11] Taylor MJ, McNicholas C, Nicolay C (2014). Systematic review of the application of the plan–do–study–act method to improve quality in healthcare. BMJ Qual Saf.

[12] Nachat A, Haroz R, LaPietra A. Opioid withdrawal in the ED: Treat or Street. ACEP Scientific Assembly. 2021.

[13] Sullivan GM, Artino AR (2013). Analyzing and interpreting data from likert-type scales. J Grad Med Educ.

